# Causes of Suicide

**Published:** 1881-06

**Authors:** 


### CAUSES OF SUICIDE.

After all there is some advantage in having a wife and children. From a comparative analysis of the statistical tables of suicides, for France and Sweden, M. Bertillon thinks he has established the following laws: (1) Widowers commit suicide more frequently than married men. (2) The existence and presence in the houses of children diminishes the inclination to suicide in both men and woman.—*Med. Press and Circular.*

